# История развития радиойодтерапии в России

**DOI:** 10.14341/probl13633

**Published:** 2025-09-14

**Authors:** М. C. Шеремета, М. В. Рейнберг, К. В. Фролов, Г. А. Мельниченко

**Affiliations:** Национальный медицинский исследовательский центр эндокринологии им. академика И.И. Дедова; I.I. Dedov Endocrinology Research Centre

**Keywords:** радиойодтерапия, история медицины, тиреотоксикоз, 131-I, щитовидная железа, radioiodine therapy, history of medicine, thyrotoxicosis, Iodine-131, thyroid gland

## Abstract

This article traces the history of the development of radioiodine therapy (RIT) as one of the leading methods for treating thyroid disorders. The relevance of the topic is determined by the high efficacy of RIT in managing thyrotoxicosis and differentiated thyroid cancer, as well as the ongoing efforts to refine dosimetric strategies and molecular imaging techniques. Based on a systematic review of historical publications and monographs from 1923 to 2022, the study analyzes key milestones in the clinical implementation of iodine-131 — from the pioneering experiments of Hertz and Seidlin in the United States in 1937 to the widespread adoption of the method in the USSR beginning in the 1950s and its subsequent advancement at the National Medical Research Center for Endocrinology of the Ministry of Health of the Russian Federation. The reviewed literature highlights the significant contributions of Russian researchers to the formation of personalized theranostic approaches and underscores the need for further improvement in planning methods, dynamic treatment monitoring, and the broader expansion of radionuclide therapies for thyroid diseases.

## МИРОВАЯ ИСТОРИЯ РАДИОЙОДТЕРАПИИ

В 1895 г. обнаружено, что щитовидная железа (ЩЖ) содержит значительное количество йода, а спустя два десятилетия экспериментально подтвердилась ее способность активно захватывать йод из циркулирующей крови [1]. Это ключевое открытие заложило основу для использования йода в диагностике и терапии заболеваний ЩЖ. В 1923 г. Henry Plummer из клиники Мейо (США) доказал эффективность назначения стабильного йода пациентам с тиреотоксикозом после операции для предотвращения тиреотоксического криза [1][2]. В том же году György Hevesy исследовал принципы применения радиоактивных индикаторов для изучения биологических процессов, хотя в то время исследования ограничивались лишь природными радиоактивными изотопами [1].

Прорыв в этой области произошел в 1934 г., когда итальянский физик Enrico Fermi в ходе экспериментов с облучением алюминиевой фольги α-частицами синтезировал 22 новых радиоактивных изотопа, включая радиоактивные изотопы йода [1][3]. Идея использования радиоактивного йода для изучения функции ЩЖ была впервые публично озвучена в 1936 г. во время доклада президента Массачусетского технологического института Karl Compton [1][4]. Вопрос о возможности применения радиоактивных изотопов йода в клинической практике задали эндокринологи Howard Means и Saul Hertz из Массачусетского многопрофильного госпиталя.

Первые эксперименты с радиоактивным йодом (I-128) были проведены в 1937 г. на кроликах. Несмотря на короткий период полураспада I-128 (25 минут), исследователям удалось подтвердить, что ЩЖ активно захватывает йод [1][4]. Для дальнейших исследований требовались более стабильные изотопы, что привело к строительству циклотрона на базе Массачусетского технологического института, который начал работу в 1940 г. С его помощью удалось получить смесь изотопов I-130 (период полураспада — 12 часов) и I-131 (период полураспада — 8 дней) [1][4]. В 1940 г. A. Roberts и S. Hertz продемонстрировали, что ЩЖ при болезни Грейвса способна захватывать до 80% введенной активности радиоактивного йода, что стало основанием для его терапевтического применения [1][5].

31 марта 1941 г. Saul Hertz провел первую в мире радиойодтерапию (РЙТ), назначив пациентке с болезнью Грейвса смесь изотопов I-130 и I-131 [4][6]. К 1943 г. им было пролечено 29 пациентов, у 20 из которых достигнут эутиреоз [4][6]. Однако из-за Второй мировой войны результаты этих исследований были опубликованы лишь в 1946 г. [1][6]. Параллельно в 1943 г. Samuel Seidlin впервые применил радиоактивный йод для лечения пациента с метастазами дифференцированного рака щитовидной железы (ДРЩЖ), что открыло новую главу в радиационной эндокринологии [1][7].

Важным этапом в развитии РЙТ стало появление в 1946 г. чистого I-131, который стал широко доступным благодаря Манхэттенскому проекту [4][8]. В 1951 г. Управление по контролю пищевых продуктов и лекарств США (FDA) официально одобрило применение I-131 для лечения заболеваний ЩЖ [1][8].

Совершенствование методов дозиметрии стало ключевым фактором в обеспечении безопасности и эффективности РЙТ. В 1940-х гг. медицинские физики Edith Quimby и Leonidas Marinelli разработали принципы расчета поглощенной дозы, учитывающие объем ЩЖ, индекс захвата I-131 и период его выведения [10][11]. В 1954 г. Международная комиссия по радиологической защите (ICRP) сформулировала принцип ALARA (As Low As Reasonably Achievable), направленный на минимизацию радиационного воздействия [10][11].

## НАЧАЛО РАЗВИТИЯ РАДИОЙОДТЕРАПИИ В РОССИИ

Развитие РЙТ в СССР началось значительно позже, чем в США и Европе, что было связано с ограниченным доступом к радиоактивным изотопам в послевоенные десятилетия. Первые систематические исследования в этой области были инициированы лишь в 1950-х гг., когда советские ученые получили возможность работать с I-131, ставшим к тому времени золотым стандартом лечения заболеваний ЩЖ [12]. С 1950 по 1954 гг. в Первом Московском медицинском институте под руководством Веры Георгиевны Спесивцевой было проведено более 200 исследований йод-захватывающей функции ЩЖ, а к 1967 г. опыт РЙТ тиреотоксикоза составил около 500 случаев с эффективностью 93,2% [12][9]. Эти работы заложили основу для клинического применения РЙТ в СССР, доказав ее эффективность у пациентов с тяжелыми формами тиреотоксикоза, для которых хирургическое вмешательство представляло высокий риск [12][13].

Несмотря на проделанные успехи, вплоть до 1980-х гг. применение РЙТ в СССР оставалось ограниченным из-за недостаточной инфраструктуры и недостаточного количества специализированных центров.

Одним из ключевых моментов в истории советской РЙТ стали 1960–1980-е годы, было открыто радиологическое отделение Московского многопрофильного научно-клинического центра им. С.П. Боткина, а также крупного специализированного отделения радиохирургического лечения открытыми радионуклидами на базе Института медицинской радиологии АМН СССР (сейчас Медицинский радиологический научный центр им. А.Ф. Цыба, г. Обнинск) [12]. Важно отметить, что вплоть до 2010 г. Обнинский центр оставался ключевым учреждением, обеспечивающим лечение пациентов со всей страны, что создавало значительные логистические сложности и ограничивало доступность РЙТ .

К 1980-м гг. РЙТ стала стандартом лечения болезни Грейвса и ДРЩЖ во многих странах. В России систематическое применение РЙТ началось в 1982 г. на базе Медицинского радиологического научного центра в Обнинске, а к 2010-му сеть специализированных центров расширилась до Москвы, Архангельска, Красноярска и других городов [12][9].

Значительный вклад в развитие радиационной эндокринологии внесли исследования А.А. Войткевича и его учеников. В рамках нового эндокринологического направления в радиационной медицине была детально изучена роль нейроэндокринной системы в патогенезе лучевой болезни. А.А. Войткевич разработал оригинальную концепцию нейроэндокринной дезинтеграции на разных стадиях заболевания, а также провел фундаментальные исследования кинетики восстановления нейросекреторных ядер гипоталамуса, клеток аденогипофиза и периферических эндокринных желез после радиационного воздействия.

Особое место в этих работах занимают исследования И.И. Дедова, проводившиеся в 1964–1972 гг. под руководством А.А. Войткевича. В лаборатории нейроэндокринологии и группе эндокринологии основное внимание уделялось изучению механизмов неспецифического адаптационного синдрома, возникающего под влиянием ионизирующего излучения. Параллельно велись поиски камбиальных и локальных стволовых клеток, способных обеспечивать регенерацию поврежденных тканей после радиационного, травматического и сочетанного поражений. Экспериментальные работы включали анализ восстановления эпителиальной, соединительной, мышечной тканей и эндокринных органов под действием гормонов и проникающей радиации.

В 1973–1982 гг. И.И. Дедов работал в лаборатории экспериментальной эндокринологии Института экспериментальной и клинической онкологии АМН СССР в Москве. В 1976 г. он защитил докторскую диссертацию «Нейроэндокринная функциональная система: онто- и филогенетические, радиационные аспекты».

В 1990-е гг. основные аспекты влияния радиационного воздействия на эндокринные органы были изложены И.И. Дедовым в книге «Радиационная эндокринология»[14]. Были предложены стохастические эффекты после аварии на Чернобыльской АЭС (ЧАЭС) на эндокринную систему, в особенности на дисфункцию щитовидной железы. Впервые было введено понятие «радиационная эндокринология» (рис. 1). Сделан вывод, что в условиях действия малых доз радиации или низкой интенсивности хронического облучения может нарушаться физиологическое течение компенсаторно-восстановительных процессов, реактивности иммунной системы, контролируемых нейроэндокринной системой, что может сказаться на жизнеспособности облученного организма, органов и тканей. Инициирована идея о необходимости изучения также нестохастических эффектов малых доз с целью определения и установления пределов доз облучения. Несмотря на наличие большого количества работ по влиянию радиации на радиочувствительные ткани (система кроветворения, герминативный эпителий), данные по влиянию на нейроэндокринную систему практически отсутствовали. Были приведены результаты острого и хронического радиационного воздействия в опытах на мелких млекопитающих, изучены последствия влияния радиации на гипоталамус, гипофиз, надпочечники, щитовидную железу, половые органы, а также эффективных доз облучения для получения описанных негативных явлений.

С 1993 г. академик И.И. Дедов совместно с О.В. Ремизовым, Н.Ю. Свириденко и рядом других специалистов принимал активное участие в реализации программы «Дети Чернобыля», направленной на диагностику и лечение заболеваний, развившихся у детей после аварии на ЧАЭС. Программа осуществлялась совместно с Московским НИИ педиатрии и в дальнейшем трансформировалась в более широкую инициативу по мониторингу здоровья детей, проживающих вблизи действующих атомных электростанций.

Начиная с 2010 г., в нескольких крупных городах, включая Москву, Архангельск, Красноярск, Челябинск и Тюмень, были открыты отделения радионуклидной терапии, оснащенные современным оборудованием [14].

Особого внимания заслуживает создание Дедовым в 2015 г. отделения ядерной медицины в ФГБУ «Эндокринологический научный центр», где был развернут отдел радионуклидной терапии и диагностики во главе с заведующим Румянцевым П.О. Событие ознаменовало новый этап в развитии терапии радиоактивным йодом. Эндокринологический центр получил возможность применения радикального лечения с использованием радиофармацевтических препаратов для пациентов с гипертиреозом и прооперированных по поводу рака ЩЖ [14]. С момента основания в отделении была развернута активная работа по диагностике заболеваний щитовидной и околощитовидных желез. В последующем спектр исследований был расширен, включив сцинтиграфию надпочечников и молекулярную визуализацию нейроэндокринных опухолей.

На начальном этапе применялись радиофармпрепараты на основе 99mTc — технетрил и пертехнетат. С 2016 г. в диагностический арсенал были включены изотонический раствор 123I и 123I-MIBG.

В отделении радионуклидной терапии основное внимание было сосредоточено на лечении пациентов с тиреотоксикозом и ДРЩЖ. С самого начала клинической деятельности была принята и реализована стратегия индивидуального дозиметрического обоснования РЙТ, что позднее стало рассматриваться как часть тераностического подхода. Уже в первые годы работы были получены значимые результаты по изучению фармакокинетики и фармакодинамики I-131, а также разработаны эффективные методы дозиметрического планирования, обеспечившие высокую клиническую эффективность и безопасность лечения.

С 2017 г. активно внедрялись технологии капсулирования радиоактивного йода и автоматизированные информационные системы поддержки принятия врачебных решений, что значительно повысило стандартизацию и точность лечебного процесса.

Ключевым фактором, определившим в России рост популярности РЙТ, стало ее включение в клинические рекомендации по лечению заболеваний щитовидной железы. В отличие от советского периода, когда данный метод применялся преимущественно в случаях, исключающих хирургическое вмешательство, современные протоколы предусматривают его использование как самостоятельный или комбинированный вариант терапии [12][14]. Например, при ДРЩЖ радиойодтерапия стала неотъемлемой частью послеоперационного лечения, направленного на абляцию остаточной тиреоидной ткани и метастазов.

Несмотря на значительный прогресс, развитие радиойодтерапии в России сталкивается с рядом вызовов. Одной из основных проблем остается неравномерное распределение специализированных центров по регионам, что ограничивает доступность метода для пациентов из удаленных районов. Кроме того, сохраняется дефицит квалифицированных кадров — медицинских физиков и радиохимиков, чья роль в планировании и проведении лечения крайне важна [14].

В заключение стоит отметить, что история радиойодтерапии в СССР и России отражает общемировые тенденции, но с характерным отставанием, обусловленным экономическими и организационными факторами. Тем не менее последние десятилетия демонстрируют устойчивую динамику роста, связанную с развитием инфраструктуры, внедрением современных стандартов и повышением квалификации специалистов. Будущее метода видится в дальнейшей персонализации лечения, основанной на точной дозиметрии и учете индивидуальных особенностей пациентов, что соответствует глобальным трендам ядерной медицины [12][14].

## ЗАКЛЮЧЕНИЕ

На протяжении последних десятилетий РЙТ в России прошла путь от экспериментального метода к высокоэффективной и научно обоснованной клинической технологии. Создание специализированных отделений, таких как Отделение радионуклидной диагностики и терапии в НМИЦ эндокринологии, позволило внедрить современные принципы тераностики, индивидуального дозиметрического планирования и молекулярной визуализации. Большой вклад в развитие РЙТ внесли отечественные специалисты в области медицинской физики, разработавшие программно-аппаратные комплексы и методики, соответствующие международным стандартам.

Сегодня одним из ключевых направлений развития является внедрение персонализированного подхода к лечению тиреотоксикоза, опирающегося не только на клинические показания, но и на фармакокинетические параметры конкретного пациента. Важно отметить, что современные европейские рекомендации (в частности, EANM и ETA) указывают на возможность раннего назначения радиойодтерапии при тиреотоксикозе без длительной предварительной медикаментозной терапии, особенно в случае рецидивирующего течения или низкой эффективности тиреостатиков. Подобный подход заслуживает более широкого распространения и в отечественной практике.

Перспективы РЙТ в России связаны с дальнейшей стандартизацией протоколов, адаптацией международных гайдлайнов, расширением применения тераностических методов, а также интеграцией информационно-технологических решений и биомаркеров для прогнозирования эффективности и безопасности лечения. Учитывая накопленный клинический опыт, кадровый потенциал и научную базу, российская школа радионуклидной терапии обладает всеми ресурсами для выхода на международный уровень в рамках персонализированной эндокринной онкологии.

## ДОПОЛНИТЕЛЬНАЯ ИНФОРМАЦИЯ

Информация о конфликте интересов. Авторы заявляют об отсутствии конфликта интересов.

Информация о финансировании. Работа по подготовке рукописи проведена в рамках государственного задания «Исследование фармакобезопасности тераностических радиофармацевтических лекарственных препаратов с использованием гибридной молекулярной визуализации в диагностике и лечении эндокринных и онкологических заболеваний в детской и взрослой возрастных группах». Регистрационный №ЕГИСУ НИОКТР 123021000041-6.

Вклад авторов. Все авторы подтверждают соответствие своего авторства международным критериям ICMJE (все авторы внесли существенный вклад в разработку концепции, проведение поисково-аналитической работы и подготовку статьи, прочли и одобрили финальную версию перед публикацией).
